# Associations between the Chronotypes and Eating Habits of Hong Kong School-Aged Children

**DOI:** 10.3390/ijerph17072583

**Published:** 2020-04-09

**Authors:** Branda Yee-Man Yu, Wing-Fai Yeung, Yuan-Shan Ho, Fiona Yan Yee Ho, Ka Fai Chung, Regina Lai Tong Lee, Mei Yuk Lam, Shucheng Chen

**Affiliations:** 1 School of Nursing, Hong Kong Polytechnic University, Hong Kong, China; branda.ym.yu@polyu.edu.hk (B.Y.-M.Y.); janice.ys.ho@polyu.edu.hk (Y.-S.H.); shucheng.chen@connect.polyu.hk (S.C.); 2 Department of Psychology, The Chinese University of Hong Kong, Hong Kong, China; fionahoyy@cuhk.edu.hk; 3 Department of Psychiatry, The University of Hong Kong, Hong Kong, China; kfchung@hku.hk; 4 School of Nursing and Midwifery, The University of Newcastle, Callaghan 2308, Australia; regina.l.lee@newcastle.edu.au; 5 School of Medical and Health Sciences, Tung Wah College, Hong Kong, China; maggielam@twc.edu.hk

**Keywords:** dietary habits, eating patterns, circadian rhythm, chrono-nutrition, morningness, eveningness

## Abstract

Later chronotypes have been found to be associated with unhealthy diets in adolescents and adults, but no study has been conducted in children. The aim of this study was to investigate the associations between the chronotypes and dietary patterns of school-aged children. Children aged 7–11 and their parents were recruited from five mainstream schools in Hong Kong. The parents were told to complete questionnaires on the children’s circadian preferences, food frequency, and dietary behaviors. All of the questionnaires were distributed and collected by schoolteachers. No gender differences in chronotype were observed (all *p* > 0.05). The evening-type was associated with significantly greater odds of viewing television (TV) during meals (adjusted odds ratios (OR) = 5.62 in boys and 5.39 in girls). Evening-oriented boys were prone to skipping breakfast (adjusted OR = 14.78), whereas evening-oriented girls were at risk of consuming fast food (adjusted OR = 7.74). There are indications of some gender differences in chronotype-related eating patterns. Sleep duration and screen time significantly mediated the associations between later chronotypes and unhealthy eating habits. Individualized dietary recommendations in accordance with circadian preferences may be effective at promoting healthy and nutritious diets for school-aged children.

## 1. Introduction

Childhood obesity has become one of the most prevalent and dramatically rising health threats to children and adolescents in recent decades, affecting an estimated 340 million individuals aged 5–19 years globally in 2016 [1]. A trend of increase in mean body mass index (BMI) has been observed in both sexes in East Asia since 2000 [2]. In Hong Kong, about 17% of primary school children were found to be overweight or obese in the 2017/2018 academic year [3]. Obese children and adolescents are at a greater risk of developing obesity and associated detrimental health consequences in adulthood, such as hypertension and diabetes [4,5,6]. Attempts have been made to identify the risk factors for childhood obesity.

Chronotype refers to an individual’s preferences with regard to the timing for engaging in sleep and other activities, which allows them to perform “at their best” [7]. Individuals are categorized into three types according to their position along a continuum between “morningness” and “eveningness”: morning-type, intermediate-type, and evening-type [8,9]. Morning-type individuals tend to have an earlier sleep-wake schedule and prefer to carry out their activities in the morning, while evening-type individuals in general sleep at a later time and function best in the late afternoon or in the evening [8]. There is increasing evidence to support the argument that there are linkages between chronotype, eating habits, and obesity in adolescents and adults [10,11].

Chronotype may play a role in predisposing an individual to follow an unfavorable dietary pattern, thereby increasing that individual’s risk of becoming obese. Arora and Taheri, in their study of 511 UK teenagers aged 11–13 years, found that evening-type adolescents were more likely to consume snacks on daily basis (*X*^2^ = 16.28, *p* = 0.012) and had an inadequate intake of fruits/vegetables (*X*^2^ = 16.45, *p* = 0.012) compared to their morning-type peers [12]. A national survey of Japanese females aged 18–20 years (*N* = 3304) conducted by Sato-Mito showed that those with the later midpoint of sleep times consumed significantly fewer vegetables (mean difference (MD) = −16.9 g/1000 kcal) and dairy products (MD = −11.8 g/1000 kcal) than those with earlier sleep times [13]. An association between circadian preference, dietary behaviors, and the likelihood of becoming overweight has been found in adolescents and young adults; however, the number of studies on these associations among individuals in younger age groups has been limited. In a study involving 2200 children and adolescents aged 9–16 years in Australia, Golley et al. examined the effect of sleep timing on diet [14]. They found that “late bed–late rise” children reported having a lower-quality diet (MD = −4.4/100 points, *p* < 0.001) than those who were “early bed–early rise”. Consistent findings were reported in the cross-sectional study by Thellman et al. on 119 American students aged 9–15 [15]. While previous studies have focused on a mixed sample of children and adolescents, studies specifically examining school-aged children are scarce The only study conducted by Chaput and colleagues on 5873 children aged 9–11 years from 12 countries confirmed the effects of bedtime on the consumption of sweetened beverages [16]. However, the study focused solely on the preference for sugar-containing beverages, and the data were only about older children. The lack of representative data may have led to some bias in our understanding of the specific features of childhood obesity and its related risk factors.

There is insufficient data on the association between chronotypes and dietary patterns in school-aged children, although circadian-related eating habits may play a crucial role in childhood obesity. Identifying circadian-related risk factors for unhealthy eating behaviors may contribute to new insights on and approaches to the early prevention of childhood obesity. In addition, most of the evidence on the impact of sleep patterns on diet has been collected in Western countries [10,12,14,15,16]. To the best of our knowledge, only a limited number of studies have been conducted in Asian populations [13,16,17]. There are known to be marked ethnic differences in dietary patterns, such as in the consumption of foods with sugar, fat, and carbohydrates [18], and also in the intake of vegetables, fruits, and snacks [19]. Cultural differences in food choices and eating habits may suggest different levels of circadian-related vulnerability to obesity in children from different cultures and have correspondingly culture-specific responses. More data on Chinese-specific dietary characteristics could possibly lead to the designing of different policies to combat childhood obesity from those in the West. Hence, our aim was to examine whether chronotypes are associated with unhealthy diets and unfavorable eating habits in Hong Kong school-aged children. Potential gender differences were also explored. Providing more up-to-date information on chronotype-related diets and eating habits will shed more light on how to devise more effective preventive strategies to respond to the rapid increase in the number of obese children today.

## 2. Materials and Methods

### 2.1. Procedure and Sampling

This is a secondary analysis of an existing dataset from the validation study of the Chinese version of Children Chronotype Questionnaire (CCTQ) in Hong Kong school-aged children [20]. Yeung et al. examined the reliability and validity of the Chinese version of the CCTQ and explored the distribution of chronotypes in Hong Kong children using a convenience sample recruited from April 2017 to July 2018. Details of the study procedures are available in the primary study [20]. The participants were children aged 7–11 years attending five mainstream primary schools in Hong Kong, as well as their parents, who had to be able to comprehend traditional Chinese characters. Questionnaires, completed by the parents, were returned by schoolteachers. Written consent to participate in the study was obtained from all the participants. The original validation study was approved by the ethical review board of the Hong Kong Polytechnic University (ref: HSEARS20170124003-01). We followed the Strengthening The Reporting of Observational studies in Epidemiology (STROBE) statement in reporting this study [21] (Supplementary Table S1). The five mainstream primary schools involved in this study had a similar start time and schedule during school days. Data collection was arranged during the semester, which avoided the exam period and long holidays. Questionnaires that were returned with any incomplete data relating to the CCTQ or that contained more than two missing items concerning eating habits were excluded. We targeted sample size to be 500, which is the recommended minimum sample size for observational studies using logistic regression (based on the Events Per Variable criterion (EPV) of 50) [22]. Considering 20% of missing data, we aimed to collect data from 600 children and their parents. Data analyses by logistic regression were conducted separately according to gender that sample calculation was also conducted for subgroup analysis. A sample of 500 is considered to be sufficient provided the result from statistical analysis in boys and girls using the formula of N = 100 + 50i, assuming no more than three indicators each group in the final model. In the end, 496 pairs of children and their parents provided sufficient data. The children were then categorized into the morning type (*n* = 54), intermediate type (*n* = 327), and evening type (*n* = 115) according to their morningness/eveningness (M/E) score as determined from the CCTQ, for further analysis.

### 2.2. Measures

#### 2.2.1. Children’s Chronotype Questionnaire (CCTQ)

The Children’s Chronotype Questionnaire (CCTQ) is a 27-item parent-reported scale for evaluating a child’s circadian preference in activities [8]. It is a valid and reliable tool for assessing chronotypes in prepubertal children aged 4–11 years. The CCTQ covers three domains: the mid-sleep point on free days (MSF), which can be derived from the reported sleep/wake parameters; the morningness/eveningness score (M/E score); and a single item of the chronotype score (CT). The mid-sleep point of free days adjusted for scheduled days (MSFsc) refers as the mid-point of sleep for free days, adjusting for sleep deficits accumulated in scheduled days. The M/E score, which ranges from 10 to 48, assesses the preferred timing of activities and the sleep/wake schedule in recent weeks. The M/E score classifies the circadian preference of children from morningness to eveningness. Those with an M/E score of below 24 points are regarded as being of the morning type (M-type); those scoring 24–32 points as the intermediate type (N-type); and those scoring at least 33 points as the evening type (E-type) [8]. The Chinese version of the CCTQ was translated and validated in our previous study [20]. The Chinese CCTQ demonstrated test–retest good reliability, with *r* = 0.52–0.86 and a Cronbach’s alpha of 0.74.

#### 2.2.2. Questionnaire on Dietary Characteristics

The size, frequency, or regularity of food behaviors are important domains to examine when studying eating behaviors [23,24], as they provide easy and convenient information with clinical significance. A dietary questionnaire modified from previous studies on dietary behaviors conducted by Stea et al. [25], Sun et al. [17], and Arora et al. [12] was therefore adopted to capture particular dietary characteristics of school-aged children. The dietary questionnaire covered some important aspects that have been widely assessed in previous studies: meal regularity and portions [13,15], snacking and fast food consumption [12,24], sugar intake [15,16], fruit and vegetable intake [12,26,27,28], and television (TV) viewing during mealtimes [13,29]. Items were prepared in English and translated to Chinese for the purpose of collecting data. For example, the question that was posed to determine whether a child had an irregular pattern of eating breakfast was “On average, how many days per week does your child have breakfast?”, with the four possible responses being “0”, “1–2 times”, “3–5 times”, and “more than 5 times”.

#### 2.2.3. Classification of Dietary Characteristics

Responses of the dietary questionnaire were then categorized as particular dietary characteristics for analysis. Although the recommended daily consumption of fruits and vegetables was 400 g or approximate five servings [30,31], recent studies have indicated that only a small percentage of children consume sufficient amounts of fruits and vegetables [32,33], therefore we categorized daily consumption of at least three portions of fruits and vegetables as more fruit/vegetable intake. Responses of “more than five times a week” to the item on the frequency of breakfast consumption were categorized as “having regular breakfast”. The responses of those children who answered “0 times a week” and “1–2 times a week” to the item on the frequency of consuming breakfast were combined with those of the children who responded “a small portion” to the item on breakfast size, and were coded as “skip breakfast/small breakfast”. A daily level of sugar consumption of less than 25 g has been advised for children aged 2–18, therefore responses of “4–6 cans weekly” and “7 cans or more a week” were categorized as consuming “over the recommended amount of sugar” [34]. Accounting for the variation of volume per unit of beverage, the average amount of sugar contained in “one can” was calculated based on 40 popular drinks to make a more accurate estimation. In addition, performing a particular activity for at least three times a week was used to refer to particular dietary behaviors that had become a habit, such as eating out, TV viewing during mealtimes, and snacking at night.

### 2.3. Statistical Analyses

The Statistical Package for the Social Sciences (SPSS) version 25.0 from IBM was used to analyze the study data. The collected data were double-entered, and the normality of the continuous data was explored using the Kolmogorov–Smirnov test. Dietary characteristics (i.e., skipping breakfast and the size of the meal consumed in the morning, greater intake of fruits or vegetables, and night-time snacking and fast-food consumption) were coded as binary data for further analyses. Due to a skewed distribution, the sleep parameters, chronotype measures, and dietary characteristics of boys and girls were compared using the non-parametric Mann–Whitney test and the Chi-square test.

Due to the known gender difference in food choices and eating habits, boys and girls were analyzed separately in order to better understand gender-specific patterns of circadian-related dietary characteristics. The Kruskal–Wallis test and the Chi-square test were used to examine differences in the median of the socio-demographic variables, MSFsc, sleep duration, and dietary characteristics of M-type, N-type, and E-type children.

Univariate logistic regressions were first conducted to determine the associations for children of both genders between dietary characteristics and a list of individual and family variables, namely: the age of the children, their birth order, their total sleep time (TST) in scheduled days and free days, screen time, family size, age and gender of their carers, marital status and employment status of their carers, level of education of their carers, and their monthly family income. Variables with a *p*-value of smaller than 0.05 were regarded as covariates and were included in a full model. Multivariate analyses were then conducted to evaluate the association between chronotypes and dietary characteristics with adjustments to those significant factors. Odds ratios (OR) with 95% confidence intervals were calculated and presented. The level of statistical significance was set at 0.05 for all analyses. The statistical analysis plan was identical for both genders, but analyses were performed separately for boys and girls.

## 3. Results

Of the 496 children who were included in the study, 52.6% of those who were interviewed were boys (*n* = 261) and their median age was 9.25 (standard deviation (SD) = 1.58) years. Most of their carers were female (78.6%), with a median age of 40 years old, without a tertiary education (73.1%), married (84.5%), and from a lower-income family (67.8%). About half of them were working parents (47.5%). The interviewed boys and girls did not differ in age or in other socio-demographic characteristics.

An overview of the sleep parameters and socio-demographic characteristics of the children is presented by gender in Table 1. With regard to the chronotypes of the boys and girls, there was no difference between them in terms of age, birth order, number of siblings, level of education of the carers, monthly family income, and so on (all *p* > 0.05).

### 3.1. Gender Differences in Chronotypes and Sleep Measures

The mid-sleep points on scheduled days and free days, and the TST on scheduled days differed significantly across chronotypes in both genders (all *p* < 0.01, Table 1). No significant difference in the distribution of chronotypes was observed between boys (M-type: 11.9%; N-type: 66.3%; E-type: 21.8%) and girls (M-type: 9.8%; N-type: 65.5%; E-type: 24.7%; all *p* > 0.05).

### 3.2. Differences between Chronotypes in Dietary Characteristics

Table 2 presents the associations in dietary characteristics and chronotypes according to gender. Boys of the E-type were found to have a lower likelihood of consuming more fruits/vegetables (10.5%), and higher likelihood of skipping breakfast/having a small breakfast (31.6%), and having a habit of viewing television during meals (73.7%) than M-type (32.3%, 3.2%, and 32.3%, respectively) and N-type boys (14.5%, 18.5%, and 50.3% respectively; all *p* < 0.05). They did not differ in other dietary characteristics.

Of the 235 girls, those of the E-type were significantly less likely to regularly have breakfast (70.7% versus 78.3% in M-type and 86.4% in N-type, *p* = 0.03), and significantly more likely to skip breakfast/have a small breakfast (44.8% versus 8.7% in M-type and 11.7% in N-type, *p* = 0.001), view TV during mealtimes (65.5% versus 17.4% in M-type and 48.7% in the N-type, *p* < 0.001), and consume excessive amounts of sugar (27.6% versus 8.7% in M-type and 11.7% in N-type, *p* = 0.01) and fast food (86.2% versus 43.5% in M-type and 79.9% in N-type, *p* < 0.001). However, there were no differences between the three groups in fruit and vegetable consumption, frequency of dining out, and habit of nighttime snacking. The multiple significant associations observed between chronotypes and dietary characteristics in both genders were further assessed with a multivariate analysis, adjusted for identified confounders.

### 3.3. Identification of Potential Confounders

We applied univariate logistics regression to explore confounding factors associated with particular dietary characteristics from a predesigned list of variables. Gender-specific associations were found between particular dietary characteristics and birth order, family size, level of education of the carers, TST in scheduled days, and screen time use. Those significant factors were included and controlled in the multivariate analyses to explore the potential associations between chronotypes and dietary characteristics (all *p* < 0.05, Table 3).

### 3.4. Associations among Chronotypes and Dietary Characteristics in Boys

Table 3 shows that the children in the three chronotype groups did not differ in terms of having a regular breakfast, excessive sugar intake, and nighttime snacking (all *p* > 0.05). E-type children seemed to eat out at night more frequently than M-type children, but did not differ much from their N-type peers in this regard (Table 2).

In the multivariate analyses (Table 3), being an N-type or E-type child was associated with less of a likelihood to consume more fruits/vegetables than being an M-type child (OR = 0.36, 95% confidence interval (CI) = 0.15 to 0.84, *p* = 0.02; OR = 0.25, 95% CI = 0.08 to 0.77, *p* = 0.02). However, the association only remained significant for N-type children after adjusting for TST-scheduled days (adjusted OR = 0.39, 95% CI = 0.14 to 0.94, *p* = 0.04), not for E-type children. A similar result was found with regard to weekly fast food consumption. E-type boys were more likely to eat fast food than M-type boys (OR = 3.62, 95% CI = 1.07 to 12.26, *p* = 0.03) but the difference did not reach a statistically significant level when adjusted for the screen time of the children (adjusted OR = 3.18, 95% CI = 0.91 to 11.16, *p* = 0.07).

Moreover, the E-type was associated with a higher proportion of those skipping breakfast/having a small breakfast (OR = 13.85, 95% CI = 1.75 to 109.64, *p* = 0.01), and the adjusted odds were even higher after controlling for the birth order of the children and their family size (adjusted OR = 14.78, 95% CI = 1.83 to 119.14, *p* = 0.01). It was also consistently found, with and without adjusting for the screen time of the children and the level of education of their carers, that E-type children were more likely to watch TV during meals (adjusted OR = 5.62, 95% CI = 1.92 to 16.42, *p* = 0.002). However, N-type boys did not differ significantly from M-type boys in their likelihood of skipping breakfast/having a small breakfast and viewing TV during meals (all *p* > 0.05).

### 3.5. Associations among Chronotypes and Dietary Characteristics in Girls

The multivariate model revealed that there was no difference between girls of different chronotypes in the consumption of fruits/vegetables, regularity of eating breakfast, frequency of dining out, excessiveness of sugar intake, and habit of nighttime snacking (all *p* > 0.05, Table 3). The E-type girls were observed to be more likely to watch TV during meals compared with their M-type peers (OR = 9.03, 95% CI = 2.70 to 30.16, *p* < 0.001). The association decreased slightly but remained significant even after adjusting for the screen time of the children and the level of education of their carers (adjusted OR = 5.39, 95% CI = 1.51 to 19.24, *p* = 0.01). N-type girls initially displayed a pattern resembling that of their E-type peers (OR = 4.51, 95% CI = 1.47 to 13.87, *p* < 0.009), but the correlation became statistically insignificant when these two confounders were taken into consideration. In addition, greater odds of weekly fast food consumption were associated with the later chronotypes even after controlling for the screen time of the children (adjusted OR = 5.50, 95% CI = 2.05 to 14.80; *p* = 0.001 in N-type; adjusted OR = 7.74, 95% CI = 2.37 to 25.24; *p* = 0.001 in E-type).

E-type girls skipped breakfast/had a small breakfast more often (OR = 8.13, 95% CI = 2.67 to 24.71; *p* < 0.001) than did girls of other chronotypes; while no significant correlation was identified in N-type girls. Nevertheless, the multivariate model did not report a significant association between E-type girls and the tendency to skip breakfast/have a small breakfast (adjusted OR = 7.95, 95% CI = 0.95 to 66.68, *p* = 0.06) when adjusted for the screen time and TST-scheduled days of the children.

## 4. Discussion

The aim of the present study was to determine the associations between chronotype and specific dietary characteristics of Hong Kong children aged 7–11. To the best of our knowledge, this is the first study to have been conducted on the link between chronotype and the eating habits of school-aged children. Circadian-related differences in eating habits were found and different patterns were observed in boys and girls, although no obvious gender difference in chronotype was identified. Children of both genders with the evening-chronotype had more of a tendency to watch TV during mealtimes than their morning-type peers. Additionally, evening-type boys were more likely to skip breakfast/have a small breakfast, while girls with the later chronotype were prone to consuming fast food on a weekly basis.

### 4.1. Gender Differences in Chronotype

Previous meta-analyses of data synthesized from studies of adolescents and adults indicated that males were more evening-oriented than females [35,36]. The data on gender differences in circadian preference among school-aged children is limited. A study of 248 children aged 8–12 in Spain provided contradictory results from studies of adolescents and adults, finding that girls were more evening-oriented than boys [37]. Children with different chronotypes had different mid-sleep points and total sleep times on school days, regardless of gender. Nonetheless, the finding that girls made up a similar proportion of the evening-type as boys is inconsistent with the results of previous studies. Due to the limited data available on school-age children, any finding on a potential gender difference in the development of chronotypes in childhood remains inconclusive.

### 4.2. Chronotype and Viewing TV during Meals

Viewing TV during mealtimes is linked with childhood obesity [38]. A recent review synthesizing data from 46 studies also indicated that there was an association between eating with the TV on and a lower quality of diet in children, including consuming less fruits and vegetables, eating more snacks and fast food, and drinking more beverages containing sugar [39]. Limiting the watching of television during mealtimes helps to prevent childhood obesity [38].

Data on the association between watching TV and chronotype is limited. Only one such study has been conducted, involving a sample of 112 Japanese female adults, where it was found that women with a later mid-sleep point were more likely to watch TV at mealtimes [13]. As mentioned previously, in the present study evening-type children were found to be more prone to watching TV at mealtimes than children with earlier chronotypes, regardless of gender. It is interesting to note that the association between the evening-type and more frequent viewing of TV during mealtimes remained significant in both genders after controlling for the level of education of the carers and the screen time of the children. Less-educated parents and longer screen time use are known predictors of viewing TV during mealtimes [39,40]. Chronotype may serve as an independent predictor from our data, and the results suggest that evening-type children are particularly vulnerable to following an unhealthy diet and are at a greater risk of becoming obese than children of other chronotypes. Understanding such chronotype-related susceptibility to viewing TV during meals highlights the importance of limiting the watching of television at mealtimes, apart from following a healthier diet.

### 4.3. Chronotype and Breakfast Consumption

Eating breakfast is known to be important for a child’s development due to the nutritional needs required for brain development and for physical/psychological health [41,42] and also its impact on school performance [43,44]. For adolescents and adults, eveningness has been associated with a greater tendency to skip breakfast [45], whereas the regular consumption of breakfast has been more commonly observed in females with an earlier chronotype [46]. The only unresolved question is whether we can identify the same trend in children.

The sole relevant study was conducted by Roßbach et al., which investigated the association between chronotype and skipping breakfast in a mixed sample of 223 individuals aged 10–18 in Germany [47]. Skipping breakfast was more often observed in children and adolescents with a later chronotype. The current study is hence the first to explore the influence of individual chronotypes on skipping breakfast in school-aged children. Evening-type children were more prone to skipping breakfast than children of earlier chronotypes. It is possible that evening-type children experience circadian misalignment to eating an early breakfast on school days, contributing to less appetite in the early morning and consequently to skipping breakfast or to consuming small portions at breakfast. Individuals with different chronotypes were also found to have unique timing-related eating patterns [48,49]. The literature provides evidence for this assumption that the food intake behaviors of evening-type people shift towards later mealtimes in accordance with their later circadian preference compared with morning-type individuals [46,47,50]. However, the association between morning-type and skipping breakfast/having a small breakfast was no longer different in girls when total sleep time on school days was taken into account. There are indications that girls have higher sleep needs than boys [51,52], therefore the effect of total sleep time may be more decisive than an individual’s circadian preference for consuming breakfast. The skipping of breakfast or lowered appetite caused by the circadian mismatch between chronotype and external schedule is hence more salient in boys.

### 4.4. Chronotype and Food Choices

#### Fruit and Vegetable Consumption

A balanced diet with a sufficient intake of fruits and vegetables is recommended to promote health and in being a protective factor against health problems, such as obesity, heart disease, and particular types of cancer [30,53]. Unfortunately, compliance with such a recommendation tends to be relatively low.

It is suggested that chronotype is a risk factor for the inadequate intake of fruits and vegetables. Most previous studies, which were conducted mainly in adults, indicated that evening-type individuals were prone to eating fewer fruits and vegetables [12,26,27,28], but gender differences were not explored. Maukonen et al. reported inconsistent results when examining the association between chronotype and fruit/vegetable consumption separately by sex [11], finding a significant difference between chronotypes only in the vegetable consumption habits of males.

We obtained similar findings as those of Maukonen et al. [11] with regard to the observation that morning-type boys were likely to consume more fruits and vegetables than boys with later chronotypes, whereas there was no difference with the girls; however, the association between evening-type and lower fruit and vegetable consumption eventually became insignificant when taking into account the total sleep time on school days. Although total sleep time on school days may be more influential than chronotype on fruit/vegetable intake, the current study pinpoints the seeming existence of a gender difference. Girls at all ages were found to consume more fruits and vegetables than boys [54]; the effect of chronotype on their fruit/vegetable intake may therefore be weakened. In light of the persistent concerns over the low intake of fruits and vegetables in children, attention should be paid to devising more individualized interventions to promote a healthy and nutritious diet for boys with a later chronotype.

### 4.5. Strengths and Limitations

This study provides insightful information to fill the gap in the existing literature on chrono-nutrition, where the previous data have mainly been drawn from adolescents and adults. A recent clinical trial examined the effectiveness of a chronotype-adjusted diet for weight control, compared to traditional dietary recommendations [55]. It was then hypothesized that evening-type individuals would benefit from dietary patterns revised in accordance with their biological rhythms. In response to the vulnerability of unhealthy eating habits associated with biological rhythms, a nutritional guideline covering the timing of eating may have additional benefits in regulating children’s appetite although it is pending for further investigation. We are the first to explore the association between chronotype and eating patterns in children; therefore, our results may contribute to the development of more effective strategies for promoting healthier dietary patterns and food choices, taking into consideration the impact of individual circadian preferences.

We acknowledge there are limitations to this study. The main limitation is the lack of nutritional analyses about food intake and lack of an objective sleep measurement. It is suggested that in a future study a food log be added to record the timing and quantity of the intake of food, enabling the nutritional contents of meals and the energy balance to be calculated. Using a validated and more comprehensive dietary questionnaire, such as the Food Frequency Questionnaire from the Children’s Eating Habits Questionnaire (CEHQ-FFQ) [56], is also recommended in future studies. Moreover, the portion sizes in the breakfasts that were consumed were not clearly indicated in the dietary questionnaire, meanwhile, the various sizes per serving of fruits and vegetables were provided with clear instructions. This may have created some ambiguities in the reporting of breakfast sizes. Another concern is the lack of an objective sleep measurement, e.g., actigraphy. Sleep duration is known to have an impact on nutrient intake [57,58] that adopting objective measures allows more precise estimation of sleep/wake pattern and sleep parameters in studying the impact of chronotype on associating sleep and dietary characteristics in children. Due to the accuracy of assessing children’s performance in school time, it would be important to involve different parties, e.g., teacher, in assessing children’s timing preference of best performance. A further consideration is parental factors, which are known to be determinants of children’s dietary patterns. Previous studies indicated that food practices [59], health concerns and parental BMI [60], and parenting styles [61,62] were associated with unhealthy dietary outcomes in children. Food environment at home and school are critical in the diet quality for school-aged children as their eating pattern are not self-sufficient [60,63,64]. Unfortunately, this study did not cover these factors, which may be a focus of further studies. Finally, the generalizability of our results is limited by single regional data collected from a Chinese population in Hong Kong, and hence replicating this study in other populations are imperative in future.

## 5. Conclusions

This study found that there were differences in the patterns of chronotype-related eating habits in boys and girls. A later chronotype may predispose an individual to follow an unhealthy diet and exhibit unfavorable dietary behaviors. Attention should be paid to the issues of skipping breakfast, consuming insufficient quantities of fruits and vegetables, viewing TV during meals, and consuming fast food in evening-oriented school-aged children. It has been noticed that sleep duration on school days and the screen time of children play significant roles in mediating the association between chronotype and eating habits.

## Figures and Tables

**Table 1:** Socio-Demographic information of the included children and their families, by sex (*N* = 496).

	Boys (*n* = 261)				Girls (*n* = 235)			
	M-Type (*n* = 31)	N-Type (*n* = 173)	E-Type (*n* = 57)	*p*-Value	M-Type (*n* = 23)	N-Type (*n* = 154)	E-Type (*n* = 58)	*p*-Value
**Children**								
**Age, yr**	9.5 ± 1.0	9.1 ± 1.1	9.4 ± 0.9	0.13	9.0 ± 1.0	9.3 ± 1.1	9.4 ± 1.1	0.31
**First-born child (*n* = 489)**	18 (58.1)	107 (62.2)	36 (63.2)	0.89	14 (63.6)	96 (64.4)	38 (65.5)	0.98
**Mid-sleep point**								
**Scheduled days**	2:02 ± 0:35	2:32 ± 0:35	2:52 ± 0:32	<0.001	2:05 ± 0:37	2:32 ± 0:35	2:51 ± 0:33	<0.001
**Free days**	2:50 ± 1:13	3:41 ± 0:52	4:15 ± 1:04	<0.001	2:57 ± 0:45	3:50 ± 0:52	4:31 ± 1:10	<0.001
**Total sleep time (mins)**								
**Scheduled days**	550 ± 35	530 ± 50	510 ± 54	<0.001	540 ± 48	520 ± 45	495 ± 53	0.001
**Free days**	580 ± 80	580 ± 70	605 ± 73	0.43	585 ± 80	592.5 ± 80	607.5 ± 83	0.08
***Their families***								
**Only child**	11 (35.5)	61 (35.3)	18 (31.6)	0.87	5 (21.7)	46 (29.9)	14 (24.1)	0.57
**Gender of carers (*n* = 491)**								
**Male**	6 (20.0)	36 (20.9)	12 (21.4)	0.99	5 (22.7)	37 (24.2)	9 (15.5)	0.40
**Female**	24 (80.0)	136 (79.1)	44 (78.6)		17 (77.3)	116 (75.8)	49 (84.5)	
**Age of carers, yr (*n* = 473)**	43.3 ± 7.9	40.4 ± 7.0	41.5 ± 6.2	0.09	40.9 ± 6.3	40.6 ± 6.1	39.5 ± 6.4	0.47
**Marital status (*n* = 483)**								
**Married**	24 (80.0)	144 (85.2)	46 (85.2)	0.76	19 (86.4)	127 (84.1)	48 (84.2)	0.96
**Single-parent**	6 (20.0)	25 (14.8)	8 (14.8)		3 (13.6)	24 (15.9)	9 (15.8)	
**Education level of carers (*n* = 480)**								
**Secondary education or below**	20 (66.7)	122 (73.5)	39 (70.9)	0.73	12 (54.5)	115 (76.7)	43 (75.4)	0.08
**University**	10 (33.3)	44 (26.5)	16 (29.1)		10 (45.5)	35 (23.3)	14 (24.6)	
**Employment status (*n* = 480)**								
**Employed**	10 (34.5)	74 (43.8)	29 (52.7)	0.26	11 (52.4)	80 (53.7)	24 (42.1)	0.33
**Not employed**	19 (65.5)	95 (56.2)	26 (47.3)		10 (47.6)	69 (46.3)	33 (57.9)	
**Family income (*n* = 472)**								
**Less than $25,000**	17 (56.7)	121 (72.9)	31 (56.4)	0.10	15 (83.3)	101 (68.7)	35 (62.5)	0.19
**$25,000–$50,000**	8 (26.7)	33 (19.9)	17 (30.9)		0 (0.0)	32 (21.8)	14 (25.0)	
**More than $50,000**	5 (16.7)	12 (7.2)	7 (12.7)		3 (16.7)	14 (9.5)	7 (12.5)	

M-type, morning-type; N-type, intermediate-type; E-type, evening-type. Data were presented as median ± inter-quartile range or number (percentage) and were compared using the Kruskal–Wallis test or chi-square test.

**Table 2:** The Differences in specific eating habits across chronotypes.

	Boys (*n* = 261)				Girls (*n* = 235)			
	M-Type (*n* = 31)	N-Type (*n* = 173)	E-Type (*n* = 57)	*p*-Value	M-Type (*n* = 23)	N-Type (*n* = 154)	E-Type (*n* = 58)	*p*-Value
More consumption of fruits & vegetables ^#^	10 (32.3)	25 (14.5)	6 (10.5)	0.02	6 (26.1)	35 (22.7)	11 (19.3)	0.78
Regular breakfast	28 (90.3)	139 (80.3)	43 (75.4)	0.24	18 (78.3)	133 (86.4)	41 (70.7)	0.03
Skip breakfast/ small breakfast	1 (3.2)	32 (18.5)	18 (31.6)	0.005	2 (8.7)	18 (11.7)	26 (44.8)	0.001
Habit of going out for dinner	0 (0.0)	18 (10.4)	7 (12.3)	0.14	4 (17.4)	26 (16.9)	8 (13.8)	0.85
Tv viewing during mealtimes	10 (32.3)	87 (50.3)	42 (73.7)	<0.001	4 (17.4)	75 (48.7)	38 (65.5)	<0.001
Excess consumption of sugar	2 (6.5)	21 (12.1)	12 (21.1)	0.11	2 (8.7)	18 (11.7)	16 (27.6)	0.01
Fast food consumption	23 (74.2)	140 (80.9)	52 (91.2)	0.09	10 (43.5)	123 (79.9)	50 (86.2)	<0.001
Nighttime snack consumption ^#^	9 (29.0)	32 (18.7)	18 (32.1)	0.08	9 (40.9)	38 (24.7)	18 (31.6)	0.22

M-type, morning-type; N-type, intermediate-type; E-type, evening-type. Data were presented as median ± interquartile range or number (percentage) and were compared using a Chi-Square test. ^#^ There were 234 girls who reported their fruit and vegetable intake and 258 boys and 233 girls who responded to the item on nighttime snack consumption.

**Table 3:** Factors associated with specific dietary variables by multivariate logistic analysis in boys and girls ^#^.

Boys			Girls		
	Adjusted OR (95% CI)	*p*-Value		Adjusted OR (95% CI)	*p*-Value
*More consumption of fruits and vegetables*					
Chronotype			Chronotype ^Ψ^		
N-type	0.39 (0.14, 0.94)	0.04	N-type	0.83 (0.31, 2.28)	0.72
E-type	0.32 (0.10, 1.04)	0.06	E-type	0.68 (0.22, 2.12)	0.50
TST-Sch D	1.01 (1.00, 1.02)	0.10			
*Regular breakfast*					
Chronotype ^Ψ^			Chronotype		
N-type	0.44 (0.13, 1.53)	0.20	N-type	1.07 (0.22, 5.23)	0.93
E-type	0.33 (0.09, 1.25)	0.10	E-type	0.47 (0.09, 2.44)	0.37
			Long screen time ^^^	0.35 (0.15, 0.81)	0.01
*Skip breakfast/small breakfast*					
Chronotype			Chronotype		
N-type	6.68 (0.87, 51.45)	0.07	N-type	1.71 (0.21, 14.12)	0.62
E-type	14.78 (1.83, 119.14)	0.01	E-type	7.95 (0.95, 66.68)	0.06
First-born child	2.82 (1.15, 6.92)	0.02	Long screen time ^^^	2.46 (1.06, 5.70)	0.04
Only child	1.45 (0.69, 3.06)	0.33	TST-Sch D	0.99 (0.98, 1.00)	0.002
*Dining out*					
Chronotype *			Chronotype ^Ψ^		
N-type	N/A	N/A	N-type	0.97 (0.30, 3.07)	0.95
E-type	N/A	N/A	E-type	0.76 (0.21, 2.82)	0.68
*TV viewing during meal times*					
Chronotype			Chronotype		
N-type	1.66 (0.68, 4.09)	0.27	N-type	2.79 (0.85, 9.12)	0.09
E-type	5.62 (1.92, 16.42)	0.002	E-type	5.39 (0.27, 0.99)	0.01
More educated carers	0.45 (0.24, 0.82)	0.01	More educated carers	0.51 (0.27, 0.99)	0.048
Long screen time ^^^	2.45 (1.41, 4.26)	0.002	Long screen time ^^^	2.15 (1.21, 3.81)	0.009
*Excessive consumption of sugar*					
Chronotype ^Ψ^			Chronotype		
N-type	2.00 (0.45, 9.01)	0.37	N-type	0.91 (0.19, 4.46)	0.91
E-type	3.87 (0.81, 18.55)	0.09	E-type	2.05 (0.39, 10.70)	0.40
			Long screen Time ^^^	3.51 (1.43, 8.61)	0.006
			TST-Sch D	1.00 (0.99, 1.00)	1.00
*Fast food consumption*					
Chronotype			Chronotype		
N-type	1.55 (0.61, 3.92)	0.36	N-type	5.50 (2.05, 14.80)	0.001
E-type	3.18 (0.91, 11.16)	0.07	E-type	7.74 (2.37, 25.24)	0.001
Long screen time ^^^	2.44 (1.22, 4.89)	0.01	Long screen Time ^^^	1.67 (0.86, 3.26)	0.13
*Nighttime snacking*					
Chronotype ^Ψ^			Chronotype		
N-type	0.56 (0.24, 1.34)	0.19	N-type	0.43 (0.17, 1.10)	0.08
E-type	1.16 (0.45, 3.02)	0.76	E-type	0.50 (0.17, 1.45)	0.20
			TST-Sch D	0.99 (0.99, 1.00)	0.03

OR, Odds ratio; CI, Confidence interval; N-type, intermediate-type; E-type, evening-type; TST-Sch D, Total sleep time in scheduled days. ^#^ Only significant covariates were presented and controlled in the multivariate analyses. ^Ψ^ Presented univariate analyses because there was no identified confounder. ^^^ Outliers were excluded from the analyses. * Analysis could not be performed because one of the cells was reported to be 0.

## Supplementary Materials

The following are available online at https://www.mdpi.com/1660-4601/17/7/2583/s1, Table S1: STROBE Statement—Checklist of items that should be included in reports of cross-sectional studies.
